# Number of children and social contacts among older people: the moderating role of filial norms and social policies

**DOI:** 10.1007/s10433-018-0469-0

**Published:** 2018-03-31

**Authors:** Anna Baranowska-Rataj, Anita Abramowska-Kmon

**Affiliations:** 1 Department of Sociology, Umeå University, Umeå, Sweden; 2 Institute of Statistics and Demography, Warsaw School of Economics, Warsaw, Poland

**Keywords:** Social contacts, Older people, Intergenerational relations, Family size

## Abstract

Social contacts offer opportunities for provision of emotional and instrumental support that enhances well-being throughout the life course, and the importance of these contacts is especially evident at advanced ages. In this paper, we take a cross-country comparative perspective to examine the association between the number of children and the frequency of social contacts among older people. Using data from the European Quality of Life Survey, we employ multilevel models with cross-level interactions between the number of children and macro-level indicators of filial norms and social policies supporting older people. Our results suggest that older adults with children are more likely than older adults without children to have frequent social interactions, but that the number of children does not affect social contact frequency. The magnitude of the association between having children and social contact frequency varies across European societies. The social contact frequency gap between older adults with children and older adults without children is larger in more familialistic countries with strong filial norms. Our results do not confirm that having children affects social contact frequency less in countries where the state provides more support for older people.

## Background

Children provide people with forms of support that enhance their physical and emotional well-being throughout their life course, which is especially crucial at advanced ages (Albertini and Mencarini 2014; Litwin and Stoeckel 2013; Litwin et al. 2015; Shiovitz-Ezra and Litwin 2015). Currently, a parent–child relationship may last as long as 50–60 years (Antonucci et al. 2013). But since European families have declined in size, older people may have fewer close family members with whom they can interact socially (Frejka 2008). Thus, demographic processes have altered the conditions shaping intergenerational relations, and especially social contact patterns among older people. The aim of this paper is to examine whether the number of children affects social contact frequency among older adults and whether the role of the number of children for social contact frequency differs depending on the societal and institutional context.

Previous research has argued that childlessness and declining fertility may contribute to the lack of social contacts among older people (Kohli et al. 2009; Reher and Requena 2017). Grundy and Read (2012) showed that having no children decreases chances of any weekly face-to-face social contact among older people. Grundy and Read (2012) additionally found that while the advantage of having children is evident at older ages, having a larger family has only a minor additional effect on the probability of having frequent social contact at older ages. However, some studies have challenged the assumption that older adults with few or no children are worse off. For example, Deindl and Brandt (2017) showed that older adults without children are often supported by their extended family, friends, and neighbours and thus that their lack of offspring is compensated for within their social network. The effect of the number of children also appears to vary across countries. While Tomassini et al. (2004) reported that parents in Italy with 3+ children had greater chances of having frequent social contact than their counterparts with 1–2 children, no such positive association was found in Finland or in the UK.

Social relations are embedded in larger social structures, most importantly, in cultural frames and institutional rules (de Jong Gierveld and Tesch-Römer 2012; Hagestad and Dykstra 2016). While studies on intergenerational relations have highlighted the role of societal norms and institutional settings (Daatland and Lowenstein 2005; Kohli et al. 2009; Lowenstein and Daatland 2006), few studies on the impact of the number of children on social contact patterns among older people have taken a cross-country comparative perspective. Systematic analyses using data for a large number of countries to examine the specific dimensions of macro-level settings that moderate the effects of the number of children on social contact frequency are missing in the literature. Our study aims to fill in this gap. We use European Quality of Life Survey data covering 24 countries (Austria, Belgium, Bulgaria, the Czech Republic, Germany, Estonia, Greece, Spain, Finland, France, Hungary, Ireland, Italy, Lithuania, Luxembourg, Latvia, Malta, the Netherlands, Poland, Portugal, Sweden, Slovenia, Slovakia, and the UK). Thanks to the large number of countries in our analysis, we were able to include specific indicators of social norms and state support for older people, as well as interactions between these indicators and the number of children of older adults. Using this approach, we examined whether the impact of having (many) children on social contact frequency varies according to country-specific filial norms and social policies supporting older people.

### The number of children and social contacts of older people

Among older people, having adult children plays an important role for frequency of social contacts (Grundy and Read 2012; Holmlund et al. 2013). Moreover, having raised children affects social network size and may therefore shape opportunities for social contacts (Kalmijn 2012; Song 2012). First, raising children strengthens parents’ relationships with relatives and friends (Gallagher and Gerstel 2001; Nomaguchi and Milkie 2003). These relationships are instrumental for the provision of mutual support and social exchange. Second, having children may provide parents with opportunities for interactions with neighbours and people involved in community institutions, such as kindergartens, schools, sport and entertainment associations, and religious organisations. Finally, parents tend to establish social ties with fellow parents through their children’s friends (Offer and Schneider 2007). While the mechanisms that underlie the development of parents’ social networks operate when their children are young, the social ties generated at that stage of the parents’ life course might persist over years (Bost et al. 2002; Klaus and Schnettler 2016). Relationships with friends, relatives, and neighbours may therefore provide opportunities for social contacts and prevent parents from becoming socially isolated as they age.

The arguments listed above imply that older adults with no or few children may have fewer social interactions than older adults with a large number of children. However, influential theories have challenged this idea. According to the hierarchical compensatory theory of social support, older people without children may compensate for their lack of offspring by developing stronger links with friends and other relatives (Cantor and Brennan 2000). Moreover, according to the resource dilution hypothesis, parents with a large number of children are able to give relatively little time and attention to each child (Blake 1989), which may diminish their affectional closeness to their children. In later life course stages, parent–child contact may be affected by the prior quality of their family relations. Parents who invested less in the quality of their relationships with their children when the children were young may receive less social support when the children become adults (Stuifbergen et al. 2008; Ward et al. 2009). These two theories imply that older adults with a large number of children cannot count on having more frequent social contact than older adults with few or no children. In sum, whether the number of children affects social contact levels at advanced ages remains an open question.

### Differences across societal contexts

Country-specific policies and legal provisions as well as social norms shape the conditions for older adults’ involvement in social activities. Previous literature has highlighted the role of familialisation, i.e., the strength of the legal and normative obligations that underpin intergenerational support (Reher 1998; Saraceno and Keck 2010). A country’s laws may mandate intergenerational support by imposing legal obligations on adult children to provide their parents with support and care (Dykstra and Hagestad 2016). Familialisation may also manifest itself in social norms that shift the responsibility of providing emotional and instrumental support to older people onto their adult children. Hence, the positive impact of having children—and especially a large number of children—on the social contact levels of parents may be stronger in familialistic countries. In such contexts, children may be more committed to maintaining frequent contact with their parents. In addition, since in familialistic countries family connections organise social life, it is less common in these countries to develop non-family ties through participation in group activities, events, and organisations outside of the family context (Mair 2013). It is therefore possible that in familialistic societies, the mechanisms postulated by the hierarchical compensatory theory are not at work. Hence, the disadvantage in social contact frequency of older adults without (many) children might be larger in familialistic societies.

In less familialistic societies, comprehensive social programmes may complement informal caring relations, thereby making older people less dependent on family support (Albertini et al. 2007; Kohli 1999). In countries where elderly care is institutionalised, the pressure on children to maintain frequent contact with their parents might be less strong, and the role of children in maintaining social contact might be weaker. Generous welfare state support for older people may also provide them with better opportunities for developing and maintaining social networks outside of their family (Ellwardt et al. 2014). With higher disposable income, it may become easier for older people to take part in social activities and events, and to become active in associations and organisations. Thus, older people with higher incomes may have ample opportunities for social interactions both within and outside of the family context. It could therefore be argued that the social contact frequency gap between older adults with few or no children and older adults with large families should be smaller in countries with more generous welfare state support for older people.

## Data and methods

We used data from the European Quality of Life Survey (EQLS) conducted in 2012 in 34 countries in Europe (European Foundation for the Improvement of Living and Working Conditions 2014). The original sample consisted of 43,636 respondents. However, we focused on older people by limiting the sample to individuals aged 65+. We also excluded countries for which the number of respondents in the older age group was too small to provide reliable estimates for the childless (i.e., Serbia, Kosovo, Macedonia, and Iceland) and for which the macro-level indicators were missing (Croatia, Cyprus, Denmark, Montenegro, Romania, and Turkey). This process decreased our sample size to 8356 respondents in 24 countries. Moreover, we excluded individuals for whom information on any variable required for our analyses was missing. The final sample included 8036 individuals.

The dependent variable was derived from questions on the frequency of direct face-to-face contact with family members (i.e., children, parents, siblings, and other relatives) living outside the household, friends, and neighbours. For each of these social categories of people with whom older people can potentially meet, EQLS provides information about a frequency of contacts (ranging from “Every day or almost every day” up to “Never”). We recoded these categories so that a higher score indicates a higher social contact frequency level. Then, we constructed the dependent variable by calculating the maximum value of the variables indicating the frequency of social contacts with family members, friends and neighbours. Our outcome variable has the following categories: “Every day or almost every day”, “At least once a week”, “One to three times a month”, and “Less often”. Hence, the variable represents the frequency of contact with the individuals with whom an older person interacts with most often. Contact frequency can be also seen as a proxy for the potential support for older people (Grundy and Read 2012; Tomassini et al. 2004). Both emotional and instrumental forms of support are more likely to be provided when contact is more frequent (Kalmijn and Dykstra 2006). As frequent contact may raise the awareness of family members and friends of an older person’s support needs, it may improve the person’s chances of receiving such support.

While contact frequency is viewed as an important measure, it also has some limitations, as it does not reflect the quality of social ties. The frequency of face-to-face contact may also be seen as a restrictive indicator, because there are alternative ways of contacting family members, neighbours, and friends, such as via letters, the telephone, or the Internet. Arguably, the opportunities for offering emotional and instrumental support are greater in direct face-to-face meetings than they are when such indirect contact channels are used. To check the robustness of our results, we carried out additional analysis on the frequency of contact with friends or family living outside the household by phone, the Internet, or by post. Again, this variable was coded from one to four, with a higher value indicating a higher social contact frequency level. Due to a higher proportion of missing values in the data on social contact via post and modern technologies, this sensitivity analysis is carried out on a smaller sample of 7996 individuals. The results from this analysis are presented in Table 3 in Appendix. Another limitation of our outcome variable is that it only covers social contact outside the household. Measurement of contact outside the household raises the question of whether the chances for social interaction differ between older people who live alone and those who co-reside with another person (a partner, an adult child, or another relative). Clearly, people who live alone are at much higher risk of social isolation and loneliness if their access to social contact outside the household is restricted. In order to address this question, we ran the analyses separately for older adults in single-person households and in multiperson households and looked at whether our results hold for both groups. The results from this analysis are shown in Tables 4 and 5 in Appendix.

The key explanatory variable, the number of own children, distinguishes between older adults with no children (the reference category), one child, two children, and 3+ children. The control variables include age grouped in the following way: 65–69 (reference category), 70–79, 80, and more. We also control for biological sex (coded as one for women and zero for men) and self-rated health (with very good, good, or fair health coded as one, and bad or very bad health coded as zero). We controlled for partnership status, distinguishing between individuals who were partnered (married or cohabiting, the reference category), widowed, divorced, or never married. We also included two measures of socio-economic status: educational attainment (distinguishing between elementary, lower secondary, upper secondary, and tertiary education, with elementary education as a reference category) and employment status (making distinction between individuals with and without employment coded as one and zero, respectively). Finally, we controlled for an urban or a rural place of residence (coded as one and zero, respectively).

We linked our micro-data with macro-level indicators that correspond to filial norms and institutional support for older people. Based on European Value Survey 2008 data, we calculated the country-specific proportions of respondents who agreed with the statements: “Adult children have the duty to provide long-term care for their parents even at the expense of their own well-being” and “Regardless of the qualities and faults of one’s parents, one must always love and respect them”. To account for the differences in the institutional conditions across countries, we used an indicator of legal provisions that make adult children responsible for supporting their parents from the Multilinks database (Keck et al. 2009). The indicator is coded as one if such legal provisions exist in a specific country, and is otherwise coded as zero. In addition, we used measures of the availability of cash for care, the minimum level of social security for older people, and the level of standard pensions for each country from the same source. The cash-for-care indicator takes a value of one if cash benefits for satisfying additional consumption or care needs are available for older people in a given country, and takes a value of zero if such cash benefits are not available. The indicator of minimum social security is measured as a ratio between the net income available within minimum income schemes for older people and the average net income of a production worker. The level of standard pensions is measured as a net replacement rate within the pension scheme of a given country for a pensioner who retired at age 65 and worked for 40 years while earning an average income. To make interpretation easier, the country-level indicators were normalised, i.e., they were rescaled so that the lowest observed value of an indicator was set to zero and the highest observed value was set to one. Thus, the coefficients measuring the effects of the cross-level interactions show the difference in the effect of the number of children on social contacts frequency when the macro-level indicators are at their highest value observed in our data, compared to when they are at their lowest.

The distributions of indicators measuring filial norms and institutional support for older people in European countries are presented in Table 2 in Appendix. Countries in our sample vary strongly with regard to filial norms, which are supported by 80–90% people in Portugal or Malta and at the same time are supported by less than every third person living in Sweden. Strong filial norms do not always overlap with legal obligations to support parents. For example, these regulations are not present in familialistic Bulgaria, but function in non-familialistic Netherlands. Institutional support for older people also varies across countries in our data. For example, replacement rates within minimum income schemes for older people range from 17% in Germany to 44% in Belgium. Replacement rates for standard pensions vary from 40% in Estonia to 120% in Greece.

Data from the EQLS are hierarchically structured with respondents nested in countries. We therefore used multilevel models that allow for dependence of observations within countries. Specifically, since our dependent variable is ordered, we estimated multilevel ordered logistic regression models (Raudenbush and Bryk 2002). The coefficients from these models correspond to the log odds and show whether explanatory variables increase or decrease the odds that the frequency of social contact among older people crosses progressively higher thresholds, as measured by our dependent variable. A positive value of a coefficient indicates that an explanatory variable increases the frequency of social contact, and a negative coefficient suggests a negative impact of the explanatory variable. To answer our research questions about the moderating roles of filial norms and institutional conditions, we included cross-level interactions between the number of children and the above-mentioned contextual variables.

## Results

**Fig. 1:**
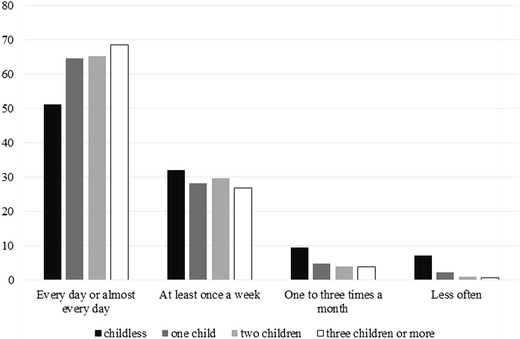
Social contact frequency among older people by number of children. *Source* EQLS 2012

For descriptive purposes, we present the frequency of social contact according to the number of children in Fig. 1. The proportion of people with fewer than one social contact per month is about 7% among the older adults without children and is 1–2% among older adults with at least one child. The proportion of people who meet someone every day or almost every day is 51% among older adults without children, and is 13–17 percentage points higher among older adults with children. Overall, this descriptive evidence suggests that having children plays an important role for the frequency of social contacts, but that the number of children does not have a large effect on the frequency of social contact.

To examine the association between the number of children and social contact frequency in a multivariate setting, and to test which specific dimensions of cross-country differences in cultural or institutional conditions moderate the impact of the number of children on social contact frequency among older people, we estimated multilevel models with cross-level interactions (see Table 1). Model 1 includes no contextual variables; Models 2 and 3 include indicators of filial norms; Model 4 introduces an indicator of legal provisions mandating that adult children are responsible for supporting their parents; and Models 5–7 include indicators of the availability of cash for care, the minimum level of social security for older people, and the standard pension level.

**Table 1:** Results from multilevel ordered logistic models of social contact frequency among older people. *Source* EQLS 2012

	Model 1		Model 2		Model 3		Model 4		Model 5		Model 6		Model 7	
	Coef.	S.E.	Coef.	S.E.	Coef.	S.E.	Coef.	S.E.	Coef.	S.E.	Coef.	S.E.	Coef.	S.E.
*Age (ref. 65–69)*														
Age 70–79	− 0.13**	(0.06)	− 0.13**	(0.06)	− 0.13**	(0.06)	− 0.13**	(0.06)	− 0.13**	(0.06)	− 0.13**	(0.06)	− 0.13**	(0.06)
Age > 80	− 0.29***	(0.07)	− 0.29***	(0.07)	− 0.29***	(0.07)	− 0.29***	(0.07)	− 0.29***	(0.07)	− 0.29***	(0.07)	− 0.30***	(0.07)
Women (ref. men)	0.16***	(0.05)	0.16***	(0.05)	0.16***	(0.05)	0.16***	(0.05)	0.15***	(0.05)	0.16***	(0.05)	0.16***	(0.05)
Good health (ref. bad health)	0.19***	(0.06)	0.20***	(0.06)	0.20***	(0.06)	0.19***	(0.06)	0.19***	(0.06)	0.19***	(0.06)	0.19***	(0.06)
*Partnership status (ref. married or cohabiting)*														
Separated	− 0.06	(0.09)	− 0.06	(0.09)	− 0.06	(0.09)	− 0.06	(0.09)	− 0.06	(0.09)	− 0.06	(0.09)	− 0.05	(0.09)
Widowed	0.21***	(0.06)	0.21***	(0.06)	0.21***	(0.06)	0.21***	(0.06)	0.21***	(0.06)	0.21***	(0.06)	0.21***	(0.06)
Never married	0.78***	(0.13)	0.78***	(0.13)	0.77***	(0.13)	0.77***	(0.13)	0.78***	(0.13)	0.78***	(0.13)	0.80***	(0.13)
*Education attainment (ref. elementary)*														
Lower secondary	− 0.19**	(0.08)	− 0.17**	(0.08)	− 0.18**	(0.08)	− 0.19**	(0.08)	− 0.19**	(0.08)	− 0.19**	(0.08)	− 0.17**	(0.08)
Upper secondary	− 0.33***	(0.08)	− 0.32***	(0.08)	− 0.33***	(0.08)	− 0.33***	(0.08)	− 0.33***	(0.08)	− 0.33***	(0.08)	− 0.32***	(0.08)
Tertiary	− 0.35***	(0.09)	− 0.33***	(0.09)	− 0.34***	(0.09)	− 0.35***	(0.09)	− 0.35***	(0.09)	− 0.35***	(0.09)	− 0.34***	(0.09)
Employed (ref. not employed)	− 0.36**	(0.16)	− 0.36**	(0.16)	− 0.36**	(0.16)	− 0.35**	(0.16)	− 0.35**	(0.16)	− 0.36**	(0.16)	− 0.35**	(0.16)
Urban area (ref. rural area)	− 0.29***	(0.05)	− 0.30***	(0.05)	− 0.29***	(0.05)	− 0.29***	(0.05)	− 0.29***	(0.05)	− 0.29***	(0.05)	− 0.29***	(0.05)
*Number of children (ref. childless)*														
1 child	1.00***	(0.09)	0.67***	(0.21)	0.64**	(0.28)	0.91***	(0.16)	1.05***	(0.16)	0.93***	(0.17)	0.50**	(0.21)
2 children	1.06***	(0.09)	0.43**	(0.19)	0.27	(0.23)	0.98***	(0.15)	1.12***	(0.15)	1.00***	(0.15)	0.68***	(0.19)
3+ children	1.13***	(0.09)	0.77***	(0.19)	0.64***	(0.24)	1.01***	(0.15)	1.18***	(0.15)	1.08***	(0.17)	0.74***	(0.21)
Filial responsibility			0.09	(0.39)										
Int. Filial resp. × 1 child			0.64*	(0.37)										
Int. Filial resp. × 2 children			1.22***	(0.32)										
Int. Filial resp. × 3+ children			0.70**	(0.33)										
Filial respect					0.11	(0.41)								
Int. Filial respect × 1 child					0.56	(0.41)								
Int. Filial respect × 2 children					1.25***	(0.34)								
Int. Filial respect × 3+ children					0.77**	(0.36)								
Legal obligations							− 0.16	(0.22)						
Int. Legal oblig. × 1 child							0.13	(0.18)						
Int. Legal oblig. × 2 children							0.11	(0.17)						
Int. Legal oblig. × 3+ children							0.17	(0.18)						
Cash for care									− 0.09	(0.23)				
Int. Cash for care × 1 child									− 0.08	(0.19)				
Int. Cash for care × 2 children									− 0.08	(0.17)				
Int. Cash for care × 3+ children									− 0.08	(0.17)				
Minimum social security for older people											− 0.43	(0.53)		
Int. Min. soc. sec. × 1 child											0.21	(0.40)		
Int. Min. soc. sec. × 2 children											0.16	(0.36)		
Int. Min. soc. sec. × 3+ children											0.16	(0.38)		
Standard pension													− 0.12	(0.48)
Int. Stand. pension × 1 child													1.17***	(0.43)
Int. Stand. pension × 2 children													0.86**	(0.38)
Int. Stand. pension × 3+ children													0.87**	(0.42)
Threshold 1	− 3.28***	(0.16)	− 3.19***	(0.26)	− 3.18***	(0.31)	− 3.39***	(0.22)	− 3.34***	(0.23)	− 3.45***	(0.27)	− 3.30***	(0.28)
Threshold 2	− 1.94***	(0.15)	− 1.86***	(0.26)	− 1.85***	(0.30)	− 2.06***	(0.21)	− 2.00***	(0.22)	− 2.11***	(0.26)	− 1.96***	(0.27)
Threshold 3	0.20	(0.14)	0.28	(0.25)	0.29	(0.30)	0.08	(0.21)	0.14	(0.22)	0.03	(0.26)	0.18	(0.27)
Variance component for intercept	0.16***	(0.05)	0.11***	(0.04)	0.11***	(0.04)	0.16***	(0.05)	0.15***	(0.05)	0.15***	(0.05)	0.14***	(0.04)
− 2 Log likelihood	− 6679.79		− 6668.36		− 6668.18		− 6679.29		− 6679.33		− 6679.44		− 6674.18	
*N*	8036		8036		8036		8036		8036		8036		8036	

*p < 0.05, **p < 0.01, ***p < 0.001, two-sided tests. Coefficients correspond to the logged odds. Parameters for thresholds differentiate the adjacent levels of the response variable, i.e., the frequency of social contact. Social contact frequency has the following categories: “Every day or almost every day”, “At least once a week”, “One to three times a month”, and “Less often”, with the higher frequency assigned to the highest value

According to the results from Model 1, social contact frequency is higher among older adults with children than among older adults without children. However, the number of children does not play a major role in social contact frequency. The Wald test of the difference between coefficients corresponding to the effects of having one child rather than two children reveals that this difference is not statistically significant, with a *p* value of 0.543. Similarly, no statistically significant differences are observed between older adults based on whether they have two children or three or more children. Overall, these results confirm earlier observations from Fig. 1 that the social contact frequency levels of older adults with children are higher than those of older adults without children, but that having a larger number of children is not associated with a higher social contact frequency level.

In the next step, we examined whether the effects of having (many) children vary across countries in ways that could be related to filial norms. Models 2 and 3 show that having at least one child increases the frequency of social contact to a greater extent in countries with stronger rather than with weaker filial norms (although this interaction is not statistically significant in Model 3 for older adults with only one child). These results suggest that the social contact frequency gap between older adults with and without children is larger in more familialistic countries.

In Model 4, we tested whether a filial legal responsibility for supporting one’s parents moderates the impact of having children on social contact frequency among older people. The results show that the presence or the absence of such regulations does not affect social contact frequency levels among older adults. More importantly, we found no evidence to support the idea that having children is associated with higher social contact frequency levels in countries where children are legally responsible for supporting their parents.

Finally, we examined whether the number of children matters less in countries where the state provides more generous financial support for older people, as measured by minimum income schemes, standard pensions, and the availability of cash-for-care benefits. The results from Models 5, 6, and 7 do not confirm this kind of buffering role of social policies. We actually found that in countries with higher standard pensions, having children has a stronger effect on the frequency of social contact than in countries where the level of financial support for older people is lower. This finding suggests that in countries where older adults have a higher disposable income, they can make more use of social networks developed through parenthood. It thus appears that welfare state support for older people reinforces rather than crowds out the support older people receive from other sources.

The results of the control variables indicate that a number of socio-economic characteristics of older people affect social contact frequency. Specifically, they show that age and poor health are associated with lower social contact frequency levels. Women tend to have more frequent social contact than men. Widowed and never-married individuals have more frequent social contact than people with a spouse or a partner. Being better educated and being employed are associated with lower social contact frequency levels. Older people living in urban areas are less likely to have at least one social contact per week than those living in rural areas.

As we mentioned in the description of our research design, we carried out additional analyses to check the robustness of our results. The development of modern communication technologies opened up new opportunities for social interaction through the telephone or the Internet. It could be argued that these forms of communication cannot entirely replace direct face-to-face contact and that they complement rather than crowd out in-person social contact among older people. Nevertheless, we checked whether the social interactions of older people through phone, the Internet, or by post display patterns similar to those of face-to-face interactions related to the number of children and the societal context. The results from this sensitivity analysis are displayed in Table 3 in Appendix. The key findings from this analysis are similar to those of our main analysis. Therefore, even if some of the social interactions of older adults take place online or by phone instead of face-to-face, our main conclusions about the role parenthood plays in different societal and institutional contexts do not change.

Our outcome variable captures social contact outside the household. This type of contact may play a very different role for older people who co-reside with another person that it does for those who live alone (Fokkema et al. 2012). As a form of additional sensitivity analysis, we ran the analyses separately for older adults in single-person households and for older people living with at least one other person (Tables 4 and 5 in ppendix). Our conclusions show that for both groups, the effect of having children depends on societal and institutional contexts in a similar way. Obviously, the meaning of the social contact patterns we observe among these two groups might differ. Nevertheless, this sensitivity analysis confirms the robustness of our main results.

## Conclusions

Previous research has shown that healthy and “successful” ageing is determined in part by social embeddedness and social contact levels (Litwin et al. 2015; Sirven and Debrand 2008). But because of ongoing demographic processes like higher childlessness rates and the trend towards smaller families, the number of close family members with whom older people can interact socially has declined (Frejka 2008). These trends could raise the risk of social isolation among older people, especially in countries where welfare state support for older people is poorly developed (Kohli et al. 2009; Reher and Requena 2017). The aim of this paper was to examine whether these processes indeed reduce social contact levels late in life and to what degree social norms or policies could moderate the disadvantages associated with not having (many) children.

Previous studies indicated that while parenthood plays an important role in the social embeddedness of older people (Grundy and Read 2012), the magnitude of the effect of having children on social contact frequency among older people may depend on the societal context (Tomassini et al. 2004; Wenger et al. 2007). Some studies have questioned whether there is an association between having (many) children and the frequency of social contacts (Albertini and Mencarini 2014; Deindl and Brandt 2017; Hank and Wagner 2013), or have challenged the idea that this relationship is universal (Wenger et al. 2007). Our results show that older adults without children have a lower social contact frequency levels than those with children. While having children reduces the risk of having no social contacts, having a larger number of children has little additional effect. The magnitude of the impact of having children on social contact frequency is, however, context-dependent. Having children may be more beneficial for older people in countries with strong filial norms as compared to less familialistic countries. According to our findings, welfare state support for older people does not diminish this gap. While it could be argued that financial support for older people may give them more opportunities for social contacts (Ellwardt et al. 2014), we find no empirical support for such a claim. More generally, our results corroborate conclusions reached in the previous research that the welfare state does not crowd out or replace family support for older adults, but rather improves the balance and structure of intergenerational relations (Daatland and Lowenstein 2005; Kohli et al. 2009; Lowenstein and Daatland 2006). We observe that in countries where older adults receive more generous support from the welfare state, the impact of having children is at least as important as it is in countries where this support is more limited.

While our study provides a number of interesting insights, it also has some limitations. The social contact frequency levels of older people may be influenced by the characteristics of the members of their social networks (Litwin et al. 2015). Unfortunately, the socio-demographic characteristics of all the individuals in the social environment of the older people are not included in our data. We also lack information on the characteristics of the children of older adults. Another limitation of our analysis is that it did not take into account the individual heterogeneity of older people in terms of both their preferences regarding their number of children and their propensity for social contact. As the number of children is not a random variable, our results may be affected by selection bias. Despite these limitations, the strength of our analysis is its international context. Cross-country comparisons combining micro- and macro-level data offer richer insights into the relationship between existing cultural and institutional settings and the situations of older people (de Jong Gierveld and Tesch-Römer 2012). Our findings extend previous research on the consequences of having no or few children by indicating under what conditions the social contact frequency gap between older adults with and without (many) children is particularly large. We show that this gap can be observed especially in familialistic countries. Previous research indicates that, paradoxically, familialistic societies are characterised by high rates of childlessness and low fertility (Reher 1998; Reher and Requena 2017). Altogether, this means that countries where having few or no children is relatively most common are also the ones where, due to social norms, a lack of (numerous) offspring has strongest impact on frequency of social contacts among older people. Social policies seem to have a limited scope for reducing this gap.

## Appendix

See Tables 2, 3, 4, and 5.

**Table 2:** Differences between institutional and cultural contexts, by country. *Sources*^a^European Value Survey 2008; ^b^Multilinks database

Country	Filial responsibility^a^	Filial respect^a^	Legal obligations to support parents^b^	Cash for care^b^	Minimum social security for older people^b^	Standard pension^b^
Austria	36.7	52.9	1	1	40.8	84
Belgium	46.8	65.3	1	1	44.3	71
Bulgaria	62.0	79.2	0	1	29.8	59
Czech Rep.	50.3	64.4	0	1	34.0	78
Estonia	48.4	68.0	0	1	18.0	40
Finland	16.4	41.7	0	1	26.2	69
France	54.6	74.4	1	1	33.8	78
Germany	39.1	58.3	1	1	16.9	64
Greece	68.1	85.2	1	1	34.1	120
Hungary	57.5	75.4	0	0	24.5	105
Ireland	43.9	62.6	0	1	38.5	84
Italy	67.1	80.8	1	1	31.1	88
Latvia	54.7	75.2	1	0	33.4	64
Lithuania	39.2	75.3	1	0	26.6	66
Luxembourg	47.8	64.1	1	1	36.9	96
Malta	79.3	90.5	1	1	38.0	79
The Netherlands	30.1	39.5	1	1	40.8	104
Poland	68.0	82.3	1	0	28.3	68
Portugal	81.4	82.4	1	1	41.4	94
Slovakia	58.5	79.1	1	1	33.9	75
Slovenia	57.1	79.4	1	0	33.2	61
Spain	67.6	75.3	1	0	25.9	95
Sweden	28.8	25.9	0	1	40.7	65
UK	37.1	59.6	0	0	27.5	73

**Table 3:** Results from multilevel ordered logistic models of social contact frequency via mail, telephone, and Internet among older people. *Source* EQLS 2012

	Model 1		Model 2		Model 3		Model 4		Model 5		Model 6		Model 7	
	Coef.	S.E.	Coef.	S.E.	Coef.	S.E.	Coef.	S.E.	Coef.	S.E.	Coef.	S.E.	Coef.	S.E.
*Number of children (ref. childless)*														
1 child	1.03***	(0.09)	0.65***	(0.20)	0.59**	(0.26)	0.94***	(0.15)	0.98***	(0.15)	1.04***	(0.16)	0.86***	(0.20)
2 children	1.06***	(0.08)	0.57***	(0.17)	0.56**	(0.22)	0.96***	(0.14)	1.05***	(0.14)	0.93***	(0.14)	0.74***	(0.18)
3+ children	1.03***	(0.08)	0.74***	(0.18)	0.68***	(0.23)	1.05***	(0.14)	1.09***	(0.14)	0.88***	(0.15)	0.84***	(0.20)
Filial responsibility			− 0.36	(0.53)										
Int. Filial resp. × 1 child			0.72**	(0.33)										
Int. Filial resp. × 2 children			0.94***	(0.29)										
Int. Filial resp. × 3+ children			0.53*	(0.30)										
Filial respect					− 0.37	(0.56)								
Int. Filial respect × 1 child					0.68*	(0.38)								
Int. Filial respect × 2 children					0.79**	(0.32)								
Int. Filial respect × 3+ children					0.55	(0.33)								
Legal obligations							0.11	(0.28)						
Int. Legal oblig. × 1 child							0.12	(0.17)						
Int. Legal oblig. × 2 children							0.14	(0.15)						
Int. Legal oblig. × 3+ children							− 0.03	(0.16)						
Cash for care									− 0.01	(0.29)				
Int. Cash for care × 1 child									0.07	(0.17)				
Int. Cash for care × 2 children									0.01	(0.16)				
Int. Cash for care × 3+ children									− 0.09	(0.16)				
Minimum social security for older people											0.84	(0.65)		
Int. Min. soc. sec. × 1 child											− 0.07	(0.38)		
Int. Min. soc. sec. × 2 children											0.36	(0.34)		
Int. Min. soc. sec. × 3+ children											0.41	(0.35)		
Standard pension													1.05*	(0.54)
Int. Stand. pension × 1 child													0.37	(0.41)
Int. Stand. pension × 2 children													0.71**	(0.36)
Int. Stand. pension × 3+ children													0.41	(0.39)
*N*	7996		7996		7996		7996		7996		7996		7996	

**p* < 0.05, ***p* < 0.01, ****p* < 0.001, two-sided tests. Coefficients correspond to the logged odds. Parameters for thresholds differentiate the adjacent levels of the response variable. Social contact frequency has the following categories: “Every day or almost every day”, “At least once a week”, “One to three times a month”, and “Less often”, with the higher frequency assigned to the highest value. Control variables as in Table 1

**Table 4:** Results from multilevel ordered logistic models of social contact frequency among older people living in single-person households. *Source* EQLS 2012

	Model 1		Model 2		Model 3		Model 4		Model 5		Model 6		Model 7	
	Coef.	S.E.	Coef.	S.E.	Coef.	S.E.	Coef.	S.E.	Coef.	S.E.	Coef.	S.E.	Coef.	S.E.
*Number of children (ref. childless)*														
1 child	0.86***	(0.13)	0.54*	(0.30)	0.59	(0.38)	0.85***	(0.22)	0.85***	(0.22)	0.83***	(0.23)	0.30	(0.27)
2 children	0.97***	(0.12)	0.35	(0.26)	0.26	(0.33)	0.88***	(0.20)	0.97***	(0.20)	0.91***	(0.21)	0.61**	(0.26)
3+ children	1.13***	(0.13)	0.61**	(0.27)	0.64*	(0.34)	0.97***	(0.22)	1.19***	(0.22)	1.01***	(0.23)	0.44	(0.29)
Filial responsibility			0.02	(0.51)										
Int. Filial resp. × 1 child			0.63	(0.53)										
Int. Filial resp. × 2 children			1.23***	(0.46)										
Int. Filial resp. × 3+ children			1.06**	(0.50)										
Filial respect					0.38	(0.52)								
Int. Filial respect × 1 child					0.42	(0.57)								
Int. Filial respect × 2 children					1.13**	(0.49)								
Int. Filial respect × 3+ children					0.78	(0.52)								
Legal obligations							− 0.20	(0.28)						
Int. Legal oblig. × 1 child							0.01	(0.25)						
Int. Legal oblig. × 2 children							0.13	(0.23)						
Int. Legal oblig. × 3+ children							0.23	(0.24)						
Cash for care									− 0.24	(0.28)				
Int. Cash for care × 1 child									0.02	(0.25)				
Int. Cash for care × 2 children									− 0.01	(0.23)				
Int. Cash for care × 3+ children									− 0.08	(0.25)				
Minimum social security for older people											− 0.67	(0.65)		
Int. Min. soc. sec. × 1 child											0.07	(0.54)		
Int. Min. soc. sec. × 2 children											0.15	(0.50)		
Int. Min. soc. sec. × 3+ children											0.31	(0.53)		
Standard pension													− 0.23	(0.61)
Int. Stand. pension × 1 child													1.34**	(0.58)
Int. Stand. pension × 2 children													0.80	(0.52)
Int. Stand. pension × 3+ children													1.54***	(0.59)
*N*	3406		3406		3406		3406		3406		3406		3406	

**p* < 0.05, ***p* < 0.01, ****p* < 0.001, two-sided tests. Coefficients correspond to the logged odds. Parameters for thresholds differentiate the adjacent levels of the response variable. Social contact frequency has the following categories: “Every day or almost every day”, “At least once a week”, “One to three times a month”, and “Less often”, with the higher frequency assigned to the highest value. Control variables as in Table 1

**Table 5:** Results from multilevel ordered logistic models of social contact frequency among older people living in multiperson households. *Source* EQLS 2012

	Model 1		Model 2		Model 3		Model 4		Model 5		Model 6		Model 7	
	Coef.	S.E.	Coef.	S.E.	Coef.	S.E.	Coef.	S.E.	Coef.	S.E.	Coef.	S.E.	Coef.	S.E.
*Number of children (ref. childless)*														
1 child	1.15***	(0.13)	0.73**	(0.32)	0.53	(0.42)	0.95***	(0.25)	1.34***	(0.25)	1.01***	(0.25)	0.67**	(0.32)
2 children	1.16***	(0.12)	0.37	(0.28)	− 0.02	(0.35)	1.04***	(0.23)	1.35***	(0.23)	1.07***	(0.23)	0.70**	(0.29)
3+ children	1.18***	(0.13)	0.76***	(0.29)	0.37	(0.37)	1.04***	(0.23)	1.34***	(0.24)	1.15***	(0.25)	0.92***	(0.32)
Filial responsibility			− 0.19	(0.50)										
Int. Filial resp. × 1 child			0.78	(0.54)										
Int. Filial resp. × 2 children			1.49***	(0.48)										
Int. Filial resp. × 3+ children			0.78	(0.49)										
Filial respect					− 0.61	(0.55)								
Int. Filial respect × 1 child					0.98	(0.61)								
Int. Filial respect × 2 children					1.85***	(0.52)								
Int. Filial respect × 3+ children					1.27**	(0.54)								
Legal obligations							− 0.23	(0.28)						
Int. Legal oblig. × 1 child							0.28	(0.29)						
Int. Legal oblig. × 2 children							0.16	(0.26)						
Int. Legal oblig. × 3+ children							0.19	(0.27)						
Cash for care									0.15	(0.29)				
Int. Cash for care × 1 child									− 0.27	(0.29)				
Int. Cash for care × 2 children									− 0.26	(0.27)				
Int. Cash for care × 3+ children									− 0.22	(0.27)				
Minimum social security for older people											− 0.40	(0.63)		
Int. Min. soc. sec. × 1 child											0.41	(0.61)		
Int. Min. soc. sec. × 2 children											0.26	(0.55)		
Int. Min. soc. sec. × 3+ children											0.10	(0.58)		
Standard pension													− 0.13	(0.61)
Int. Stand. pension × 1 child													1.08*	(0.65)
Int. Stand. pension × 2 children													1.02*	(0.58)
Int. Stand. pension × 3+ children													0.57	(0.63)
*N*	4630		4630		4630		4630		4630		4630		4630	

**p* < 0.05, ***p* < 0.01, ****p* < 0.001, two-sided tests. Coefficients correspond to the logged odds. Parameters for thresholds differentiate the adjacent levels of the response variable. Social contact frequency has the following categories: “Every day or almost every day”, “At least once a week”, “One to three times a month”, and “Less often”, with the higher frequency assigned to the highest value. Control variables as in Table 1
